# How Was the Weekend? How the Social Context Underlies Weekend Effects in Happiness and Other Emotions for US Workers

**DOI:** 10.1371/journal.pone.0145123

**Published:** 2015-12-23

**Authors:** John F. Helliwell, Shun Wang

**Affiliations:** 1 Vancouver School of Economics, University of British Columbia, Vancouver, British Columbia, Canada; 2 Canadian Institute for Advanced Research (CIFAR), Toronto, Ontario, Canada; 3 Korea Development Institute (KDI) School of Public Policy and Management, Sejong, Korea; University of Vermont, UNITED STATES

## Abstract

In this paper we estimate the size of weekend effects for seven emotions and then explore their main determinants for the working population in the United States, using the Gallup/Healthways US Daily Poll 2008–2012. We first find that weekend effects exist for all emotions, and that these effects are not explained by sample selection bias. Full-time workers have larger weekend effects than do part-time workers. We then explore the sources of weekend effects and find that workplace trust and workplace social relations, combined with differences in social time spent with family and friends, together almost fully explain the weekend effects for happiness, laughter, enjoyment and sadness, for both full-time and part-time workers, with significant but smaller proportions explained for the remaining three emotions—worry, anger and stress. Finally, we show that workplace trust and social relations significantly improve emotions and life evaluations on both weekends and weekdays for all workers.

## Introduction

Emotions, both positive and negative, are key measures of subjective well-being [1], [2], [3]. Recent empirical studies on emotions find evidence of variations through the week, often called a day-of-week effect. One study finds that those who are interviewed on Fridays report lower levels of mental stress than those interviewed in the middle of the week, using the British Household Panel Survey (BHPS) data [4]. Some studies focus on the variations between weekends and weekdays, namely the weekend effect. For example, a recent study finds that people experience more positive emotions and fewer negative emotions during weekends and statutory holidays than on weekdays in the Gallup/Heathways US Daily Poll [2]. A few other studies also find similar weekend effects [5], [6], [7], [8], [9].

Though the weekend effect seems to be well-proved, some recent studies pose challenges. Some researchers argue that the day-of-week or weekend effects appearing in some datasets might be due to possible selection bias in the choice of interview days [4], [10]. In other words, the subjective well-being responses might be correlated with some observed or unobserved characteristics that affect individuals’ decisions to take interviews on specific days. The two studies are both based on the British Household Panel Survey data. One study draws the conclusion that the day-of-week pattern of job satisfaction is not substantially affected by the potential selection bias, although the day-of-week pattern for mental well-being does become less significant after adjusting for the likely bias [11]. Another one emphasizes the potential selection bias originating from unobservables and suggests that the day-of-week patterns may vary across countries [10].

A few recent studies discover variations of weekend effects across demographic groups and social environments. One recent paper shows that weekend effects for emotions are smaller among older workers, particularly with respect to negative emotions [8]. Another one finds that weekend effects vary with gender, marriage status, age, and working status [2]. Some find that the variation of social time across days of week is an important determinant of weekend effects [2], [9]. The quality of the social context at work has previously been shown to influence both life evaluations and emotions [2], [11].

Differing from previous studies, in this paper we focus more specifically on emotional weekend effects for the full-time and part-time working population using the Gallup/Heathways Daily Poll in the United States. In this way we are able to assess more directly, for a comparable set of respondents, the size of weekend effects for each of the seven emotions (happiness, enjoyment, laughter, worry, sadness, anger, and stress). We will also explore the varying explanatory power of the social context, both on and off the job, for each emotion, comparing full-time workers with part-time workers.

Seeing the possibilities of selection bias, we also deal with the selection issue in our data. Since we are studying weekend effects rather than day-of-week effects, we are mostly concerned about whether individuals’ answers depend on whether they were interviewed on weekends or weekdays, rather than whether they are interviewed on particular days of the week, as studied in [4], [10].

## Data and Methodology

The data we use for this study come from the Gallup/Healthways US Daily Poll. From the beginning of 2008, Gallup has randomly interviewed about 1,000 American adults each day in the United States. By the end of 2012, the total number of respondents accumulated in the data is 1.77 million. The Daily Poll includes a set of questions on emotional well-being: for positive emotions we have happiness, enjoyment, and laughter, and for negative emotions we have worry, sadness, anger, and stress. Laughter is a yes or no response (yes = 1) to the question “Did you smile or laugh a lot yesterday?” Other six emotions are similar binary responses to the question “Did you experience the following feelings during a lot of the day yesterday?” Emotion questions were asked on every survey day, except for the stress question, which was not asked in 2011 and 2012.

The survey includes the labor force status for each respondent, so that we can derive a sample based on the working population. There are about 0.94 million working respondents, accounting for 58% of all respondents. Among the working respondents, nearly 80% are full-time paid workers.

The key explanatory variables are social hours and two measures of the quality of the workplace social context. The social hours variable is a response to the question “Approximately, how many hours did you spend, socially, with friends or family yesterday? Please include telephone or e-mail or other online communication.” This question was asked of all the respondents from 2008 to 2010, but in 2011 and 2012 only 10% and 5% of total respondents, randomly selected, were asked this question. Respondents report numbers between 0 and 24. Among the answers, there are about 5% of respondents reporting more than 16 social hours. To make this social hours variable more reliable, we replace any value greater than 16 by 16. There are two questions on workplace environment. One is “Does your supervisor always create an environment that is trusting and open, or not?” The answer to this question is binary, 1 for “yes” and 0 for “no”. 80% of respondents answer that the environment is trusting. Another question is “Does your supervisor at work treat you more like he or she is your boss or your partner?” The answer to this question is also binary, 1 for “partner” and 0 for “boss”. 62% report having a “partner-like” boss.

The survey also includes a number of socio-demographic variables, such as gender, age, marital status, level of education, number of children under 18, monthly household income, health insurance coverage and importance of religion. Monthly household income refers to before-tax income from all sources, including wages and salaries, remittances from family members living elsewhere, farming, and others. The response is categorical, in which zero to ten stands for no income, under $60, $60 to $499, $500 to $999, $1,000 to $1,999, $2,000 to $2,999, $3,000 to $3,999, $4,000 to $4,999, $5,000 to $7,499, $7,500 to $9,999, and $10,000 and over, respectively. We construct the numerical household income by replacing the categorical response by the mean of each non-top category, and $18,000 for the top income category. There are about 0.78 million working respondents reporting income. To reduce the impact of missing income on the number of observations, we assign a zero value to log income when income is missing, and a dummy variable which equals to 1 if the income is missing will be used together with log income in regressions. The summary statistics for all the variables are reported in Table 1.

**Table 1:** Summary Statistics of Explanatory Variables.

Variable	N	Mean	Std. Dev.	Min	Max
Male	935,010	0.540	0.498	0	1
Age	921,566	42.529	14.105	18	99
Married or living with partner	925,843	0.621	0.485	0	1
Separated, divorced, or widowed	925,843	0.137	0.344	0	1
Education					
*High school*	925,451	0.321	0.467	0	1
*Some college*	925,451	0.238	0.426	0	1
*College*	925,451	0.210	0.407	0	1
*Graduate*	925,451	0.164	0.370	0	1
Log household income	935,012	9.216	4.008	0	12.283
Dummy for zero or missing income	935,012	0.153	0.360	0	1
Church attendance					
*weekly*	905,127	0.086	0.280	0	1
*monthly*	905,127	0.123	0.328	0	1
*seldom*	905,127	0.268	0.443	0	1
*never*	905,127	0.205	0.404	0	1
Having health insurance	934,102	0.846	0.361	0	1
Number of children	933,392	0.864	1.198	0	15
Importance of religion in life	930,609	0.624	0.484	0	1
Full-time paid worker	935,012	0.797	0.402	0	1
Social time with family or friends	558,667	6.024	4.529	0	16
Dummy for zero social hour	558,667	0.038	0.191	0	1
Dummy for zero to one social hour	558,667	0.028	0.164	0	1
Dummy for non-trusting workplace	774,382	0.203	0.403	0	1
Dummy for boss-like supervisor	769,743	0.380	0.485	0	1

To show the size of weekend effects, we estimate the following model for each emotion for full-time and part-time workers separately:

$$emotion_{it}=\alpha +\beta weekend_{it}+X_{it}^{\prime }\Gamma +X_{ct}^{\prime }\Omega +\varepsilon_{it},$$ (1)

where *i* indexes individuals. The variable *emotion*_*it*_ denotes one of the seven emotions, while *weekend*_*it*_ is an indicator variable that equals to one if the emotions are for weekends or statutory holidays. The vector *X*_*it*_ denotes a set of individual- and household-level covariates, which include respondent’s gender, age, age squared divided by 100, marital status, education levels, household income, number of children, frequency of church attendance, an indicator variable that equals to one if having health insurance, and a dummy variable indicating the importance of religion in life. *X*_*ct*_ is a vector for state-year fixed effects. *ε*_*it*_ is the error term.

To see how the weekend effect is varying with each respondent’s workplace environment, we estimate the following model for full-time and part-time workers respectively:

$$emotion_{it}=\alpha +\beta weekend_{it}+\gamma weekend_{it}*work_{it}+\theta work_{it}+X_{it}^{\prime }\Gamma +X_{ct}^{\prime }\Omega +\varepsilon_{it},$$ (2)

where the variable *work*_*it*_ denotes the quality of the workplace environment. We have two measures for workplace quality: one is a dummy variable that equals to one if respondents report having a workplace is trustworthy and open, and the second is a dummy variable that equals to one if respondents report having a supervisor who is more like a partner than a boss. The two measures will first be included in the regression individually and then together. This is a typical difference-in-difference (DID) approach, where γ captures the difference in weekend effect by workplace environment.

Next we add the social time variables into Eq (2) to check how well weekend effects are further explained by social hours. The equation is as follows:

$$emotion_{it}=\alpha +\beta weekend_{it}+\gamma weekend_{it}*work_{it}+\theta work_{it}+S_{it}^{\prime }\Psi +X_{it}^{\prime }\Gamma +X_{ct}^{\prime }\Omega +\varepsilon_{it},$$ (3)

where *S*_*it*_ denotes a set of variables on social time, including log of social hours, a dummy variable for zero social hour, and a dummy for zero to one social hour.

## The Size of Weekend Effects

### Descriptive results

Table 2 gives a detailed summary of the three positive emotions—happiness, enjoyment, and laughter, and four negative emotions—worry, sadness, anger, and stress. In the table we report the number of observations, estimated means and standard errors for each emotion on weekends and weekdays, and weekend effects measured by the mean difference of each emotion between weekends and weekdays, and the percentage of change of emotion from weekdays to weekends. We can see generally that positive emotions are more prevalent than negative ones. Moreover, the prevalence of positive emotions is higher on weekends than weekdays, with the reverse applying to negative emotions. The weekend effect is statistically significant for each emotion. Moreover, the effect is sizable, with absolute values ranging from 0.014 to 0.150, and the percentage change ranging from 3.3% to 32.5%. The percentage improvements for negative emotions from weekdays to weekends are generally larger than for positive emotions, consistent with the findings in [9]. For example, the reductions in stress, worry, and anger are -32.5%, -24.3%, -24.4% respectively, much larger than the improvement in enjoyment, 6.8%, the most improved among the positive emotions. These differences primarily reflect the fact that the average frequency is much less for negative than for positive emotions.

**Table 2:** Weekend Effects on Emotions for the Working Population.

Variable	Weekends		Weekdays		Weekend Effect	
	N	Mean	N	Mean	Absolute	Relative
Happiness	296,623	0.922	636,477	0.893	0.030***	3.3%
		(0.001)		(0.001)	(0.001)	
Enjoyment	296,656	0.902	636,549	0.844	0.058***	6.8%
		(0.001)		(0.001)	(0.001)	
Laughter	295,975	0.871	634,774	0.837	0.034***	4.1%
		(0.001)		(0.001)	(0.001)	
Worry	296,878	0.248	637,188	0.328	-0.079***	-24.3%
		(0.001)		(0.001)	(0.001)	
Sadness	296,923	0.130	637,332	0.144	-0.014***	-9.6%
		(0.001)		(0.001)	(0.001)	
Anger	296,965	0.111	637,415	0.146	-0.036***	-24.4%
		(0.001)		(0.001)	(0.001)	
Stress	173,762	0.311	364,162	0.460	-0.150***	-32.5%
		(0.001)		(0.001)	(0.002)	
Ladder	288,779	6.941	643,642	6.923	0.017***	0.2%
		(0.004)		(0.003)	(0.005)	

Notes: Absolute weekend effect is the difference between emotion on weekends and weekdays. Relative weekend effect is the absolute weekend effect divided by the average value on weekdays, measured as a percentage difference. Standard errors for means and differences are reported in parentheses. +, *, **, and *** indicate significance at the 10, 5, 1, 0.1% levels respectively.

### Regression results

In this section we show the estimates of weekend effects using Eq (1). We run OLS regressions for each emotion, separately for full-time and part-time workers. Though all emotion variables are binary, we still run OLS for easier interpretation of the coefficients. However we have checked the results using logistic regressions and find similar results. For each group, we run two models, one is the model controlling only for state-year fixed effects, while the second includes state-year fixed effects and the full set of covariates described in Eq (1): gender, age, age squared divided by 100, marital status, education levels, household income, number of children, frequency of church attendance, an indicator variable that equals to one if having health insurance, and a dummy variable indicating the importance of religion in life. We report the coefficients of the weekend dummy in Table 3. We find that the weekend coefficients for each emotion are almost the same in the two models, which indicates that other covariates of weekend and weekday samples are well balanced, given the random sampling procedure. Moreover, the weekend effect for full-time workers is larger than for part-time workers. For happiness, enjoyment, anger and stress, the weekend effect for part-time workers is approximately half that for full-time workers. The relative size is two-thirds for laughter, three-quarters for worry, while almost equal for sadness.

**Table 3:** Weekend Effects for Full-Time and Part-Time Workers.

	Happiness	Enjoyment	Laughter	Worry	Sadness	Anger	Stress
*Panel A*: *Full-time paid worker*							
State-year fixed effect	0.033***	0.065***	0.037***	-0.085***	-0.014***	-0.041***	-0.168***
Full set of controls from Eq (1)	0.033***	0.065***	0.037***	-0.085***	-0.015***	-0.040***	-0.167***
*Panel B*: *Part-time paid worker*							
State-year fixed effect	0.017***	0.034***	0.026***	-0.063***	-0.013***	-0.017***	-0.087***
Full set of controls from Eq (1)	0.017***	0.034***	0.026***	-0.063***	-0.014***	-0.018***	-0.087***

Notes: Each cell of the table reports OLS estimates of the weekend effect. In one model, only state-year fixed effects are controlled. In another model, the covariates include the full set of controls from Eq (1). Panel A and B is for full-time and part-time paid worker respectively. In both regressions, the sample sizes are the same. Standard errors (not reported in the table) to calculate the significance level are clustered within counties. +, *, **, and *** indicate significance at the 10, 5, 1, 0.1% levels respectively.

### Testing for selection effects

As shown in previous studies [4], [10], the size of day-of-the-week effects may be affected by the potential bias of respondents’ self-selection into specific days within a week, for surveys where only a single day is reported for each respondent, as is the case with our data. In this section we run a few tests to show that the impact of selection bias (being self-selected into weekends versus weekdays) in our data is fairly small, if not zero. We report the results only for paid workers, but the tests for full samples yield similar results.

In Table 4 we test the balance of social-demographic variables for respondents being surveyed on weekends and weekdays. Columns (1) and (2) report the mean of each variable for respondents being surveyed on weekends and weekdays respectively. Columns (3) and (4) show the mean difference between Weekends and Weekdays and the corresponding standard error. We will find small differences some variables, some of which are rendered statistically significant by the large sample size. In this case we may check the standardized differences of means, which is a common way to check sample balance in propensity score matching methods, following [12], [13]. Column (5) reports the standardized differences of means calculated by the formula

$$\rho \left(x\right)=\frac{{\bar{x}}_{1}-{\bar{x}}_{2}}{\sqrt{0.5\left(Var\left(x_{1}\right)+Var\left(x_{2}\right)\right)}},$$ (4)

where ${\bar{x}}_{1}$ and ${\bar{x}}_{2}$ are the sample means for weekends and weekdays, and *Var*(*x*_1_) and *Var*(*x*_2_) are the corresponding sample variances. We can see that the absolute value of each standardized difference is smaller than 2.2. This is much smaller than the often-used cutoff value 10. This confirms the similarity of the weekend and weekday samples.

**Table 4:** Balancing Test.

Variable	(1)	(2)	(3)	(4)	(5)
	Weekend	Weekdays	Difference	s.e.	Standardized Difference
Male	0.539	0.540	-0.001	0.001	-0.155
Age	42.668	42.468	0.201 ***	0.039	1.424
Age squared/100	20.190	20.027	0.164 ***	0.033	1.304
Married or living with partner	0.614	0.625	-0.010 ***	0.001	-2.100
Separated, divorced or widowed	0.140	0.136	0.004 ***	0.001	1.133
Education					
*High school or vocational school degree/diploma*	0.316	0.323	-0.007 ***	0.001	-1.482
*Some college*	0.241	0.237	0.003 **	0.001	0.796
*College graduate*	0.208	0.210	-0.002	0.001	-0.409
*Post graduate work or degree*	0.169	0.161	0.008 ***	0.001	2.143
Log household income	9.265	9.195	0.070 ***	0.011	1.749
Indicator for missing income	0.149	0.155	-0.006 ***	0.001	-1.685
Church attendance					
*Weekly*	0.085	0.086	-0.001	0.001	-0.370
*Monthly*	0.124	0.122	0.002	0.001	0.469
*Seldom*	0.268	0.268	0.000	0.001	-0.012
*Never*	0.207	0.204	0.003 **	0.001	0.770
Having health insurance	0.848	0.845	0.004 **	0.001	1.006
Number of children	0.852	0.869	-0.017 ***	0.003	-1.449
Importance of religion in life	0.623	0.625	-0.002	0.001	-0.431
Full-time paid worker	0.800	0.795	0.005 ***	0.001	1.241

Notes: +, *, **, and *** indicate significance at the 10, 5, 1, 0.1% levels respectively. Standardized difference is calculated by Eq (3).

**Fig 1:**
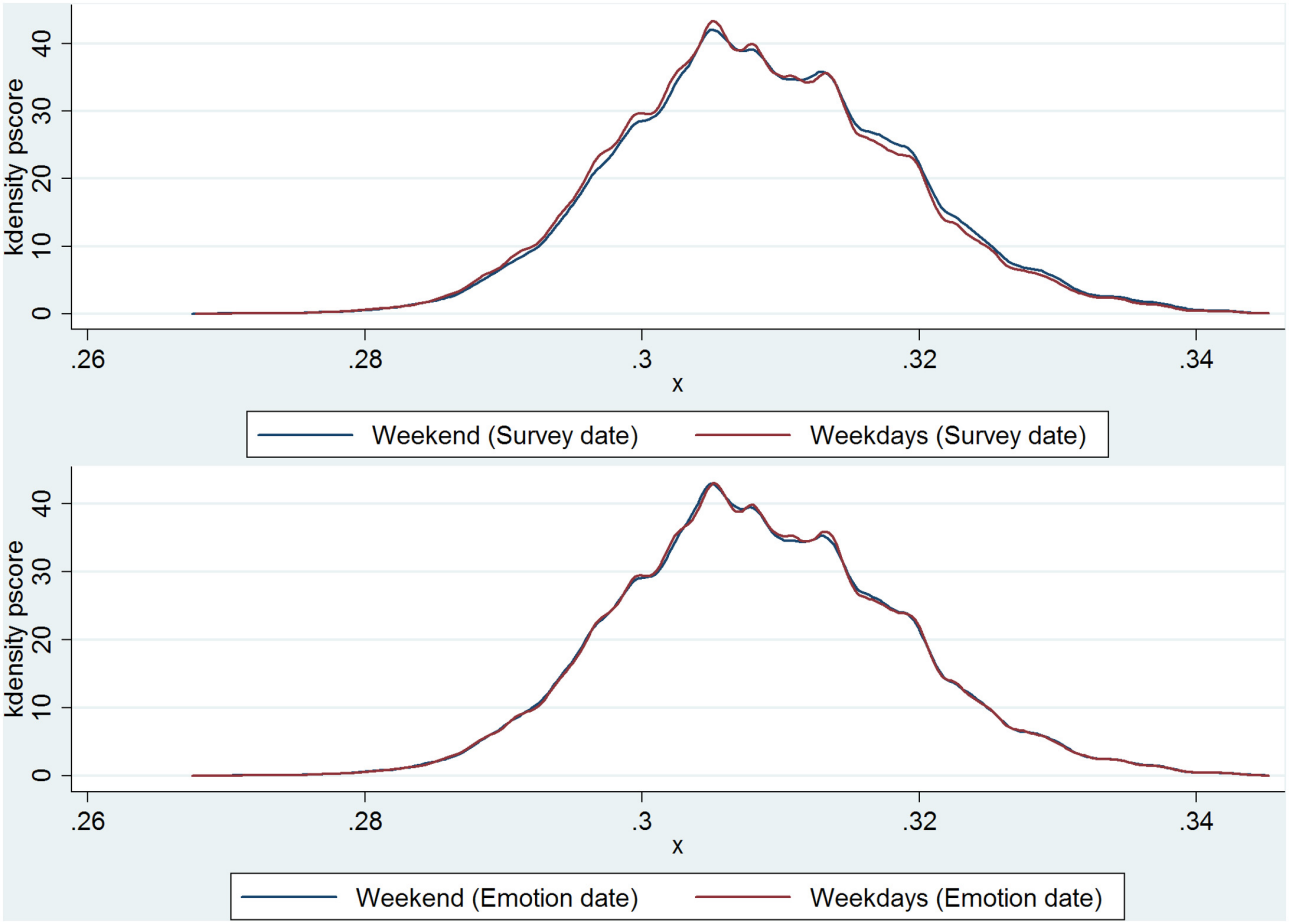
Propensity Score. Notes: The upper panel of Fig 1 shows the distribution of propensity scores for people who were actually interviewed on weekends and weekdays, and the lower panel shows the distribution of propensity scores for people who report emotions for weekends (who were interviewed on Sunday and Monday) and weekdays (who were interviewed on Tuesday to Saturday).

We also illustrate in Fig 1 the propensity scores for being selected for weekend interviews. We use a *probit* model to estimate the propensity score. The variables used to estimate the propensity score of being selected into weekend include respondent’s gender, age, age squared divided by 100, marital status, education levels, household income, number of children, frequency of church attendance, an indicator variable that equals to one if having health insurance, a dummy variable indicating the importance of religion in life, and respondent’s full-time or part-time working status. The upper panel of Fig 1 shows the distribution of propensity scores for people who were actually interviewed on weekends and weekdays, and the lower panel shows the distribution of propensity scores for people who report emotions for weekends (who were interviewed on Sunday and Monday) and weekdays (who were interviewed on Tuesday to Saturday). In both cases, the distribution of propensity scores is very similar for the two groups, which suggests that each respondent has almost the same probability of being selected into weekends or weekdays.

Next we make a direct test for selection bias by exploiting the nice feature of our data that people report yesterday’s emotions. The emotions relating to Sunday, Monday and Tuesday which are reported on Monday, Tuesday, and Wednesday, respectively, are not affected by possible weekend selection bias. Column (1) of Table 5 shows the difference in emotions between Sunday and Monday and column (2) shows the difference in emotions between Tuesday and Monday. The values in column (2) are all equal to or very close to zero, suggest that emotions are stable across weekdays. The large differences between column (1) and column (2) suggest that real weekend effects exist. Thus the weekend effect we observe is not just driven by selection effects. We then compare the weekend effect measured by the difference between emotions on Sunday and Monday in column (1), with the regular weekend effect based on all data in column (3). We know that the former is not subject to selection bias, but the latter may be subject to certain level of selection bias. However, since the two results are so similar, the bias must be negligibly small. Note that our results suggest that there is no selection between weekends and weekdays in the Gallup/Heathways US Daily Poll, which may not mean the previous argument of respondents’ self-selection into specific days within a week in the British Household Panel Survey [10] is invalid, since the type of selection bias and the nature of data are different in the two cases.

**Table 5:** Test for Selection Bias.

	(1)	(2)	(3)
	Sun-Mon	Tue-Mon	Weekend effect
Happiness	0.035***	0.001	0.030***
	(0.002)	(0.002)	(0.001)
Enjoyment	0.066***	0.001	0.058***
	(0.002)	(0.002)	(0.001)
Laughter	0.040***	0.002	0.034***
	(0.002)	(0.002)	(0.001)
Worry	-0.084***	0.004+	-0.079***
	(0.002)	(0.002)	(0.001)
Sadness	-0.015***	0.002	-0.014***
	(0.002)	(0.002)	(0.001)
Anger	-0.035***	0.007**	-0.036***
	(0.002)	(0.002)	(0.001)
Stress	-0.151***	0.015***	-0.150***
	(0.003)	(0.003)	(0.002)

Notes: The first column shows the difference in emotions between Sunday (being surveyed on Monday) and Monday (being surveyed on Tuesday). The second column shows the difference in emotions between Tuesday (being surveyed on Wednesday) and Monday (being surveyed on Tuesday). The third column shows the difference in emotions between weekends and weekdays. The emotions on Sunday, Monday and Tuesday were reported on Monday, Tuesday, and Wednesday respectively. If there is no real weekend effect, in other words, if weekend effects are solely driven by respondents’ self-selection (into weekends versus weekdays), we should not observe large difference between columns (2) and (3), because these effects are calculated from emotions reported on weekdays. Moreover, since column (1) reports weekend effects under potential selection bias (if any) while column (2) reports weekend effects without selection bias, the close similarity of the two results suggests that the section bias is minimal, if not zero. Standard errors in parentheses are clustered at counties, and +, *, **, and *** indicate significance at the 10, 5, 1, 0.1% levels respectively.

## Exploring the Determinants of Weekend Effects

In this section we explore the determinants of weekend effects. We first run OLS regressions following Eq (2) to examine how weekend effects vary with workplace social context, measured by reported workplace trust and type of supervisor (boss-like or partner-like). We further control social time variables following Eq (3). We report the estimated weekend effect for happiness in Tables 6 and 7 for full-time and part-time workers respectively. In each table, Models (1), (3) and (5) follow Eq (2) and Models (2), (4) and (6) follow Eq (3). We use the dummy for non-trusting workplace and its interaction with the weekend dummy in Models (1) and (2), the dummy for boss-like supervisor and its interaction with the weekend dummy in Models (3) and (4), and both workplace dummies and their interactions with the weekend dummy in Models (5) and (6).

**Table 6:** Determinants of Happiness (Yesterday) for Full-time Workers.

	(1)	(2)	(3)	(4)	(5)	(6)
Weekend	0.025***	0.000	0.025***	-0.000	0.022***	-0.003*
	(0.001)	(0.001)	(0.001)	(0.001)	(0.001)	(0.001)
Dummy for non-trusting workplace	-0.097***	-0.087***			-0.082***	-0.073***
	(0.002)	(0.002)			(0.002)	(0.002)
Dummy for non-trusting workplace*Weekend	0.045***	0.041***			0.039***	0.034***
	(0.003)	(0.003)			(0.003)	(0.004)
Dummy for boss-like supervisor			-0.058***	-0.055***	-0.031***	-0.030***
			(0.001)	(0.002)	(0.001)	(0.002)
Dummy for boss-like supervisor*Weekend			0.025***	0.025***	0.012***	0.014***
			(0.002)	(0.002)	(0.002)	(0.003)
Log social hours		0.053***		0.054***		0.053***
		(0.001)		(0.001)		(0.001)
Dummy for zero social hour		-0.108***		-0.109***		-0.108***
		(0.006)		(0.005)		(0.005)
Dummy for zero to one social hour		-0.057***		-0.059***		-0.057***
		(0.005)		(0.005)		(0.005)
Number of observations	611,585	371,519	608,171	369,620	603,706	366,881
Number of counties	3,123	3,116	3,123	3,116	3,123	3,116
Adjusted R-squared	0.031	0.064	0.024	0.059	0.032	0.065

Notes: The odd columns follow Eq (2) and the even columns follow Eq (3). Variables in the vector ${\text{X}}_{it}^{\prime }$ and state-year dummies are controlled in all models, but coefficients are not reported. The Standard errors in parentheses are clustered within counties. +, *, **, and *** indicate significance at the 10, 5, 1, 0.1% levels respectively.

**Table 7:** Determinants of Happiness (Yesterday) for Part-time Workers.

	(1)	(2)	(3)	(4)	(5)	(6)
Weekend	0.014***	-0.001	0.013***	-0.001	0.013***	-0.002
	(0.002)	(0.003)	(0.003)	(0.003)	(0.003)	(0.003)
Dummy for non-trusting workplace	-0.081***	-0.078***			-0.073***	-0.070***
	(0.005)	(0.006)			(0.005)	(0.006)
Dummy for non-trusting workplace*Weekend	0.010	0.018+			0.010	0.017
	(0.007)	(0.009)			(0.008)	(0.010)
Dummy for boss-like supervisor			-0.037***	-0.036***	-0.019***	-0.017***
			(0.003)	(0.004)	(0.003)	(0.004)
Dummy for boss-like supervisor*Weekend			0.006	0.010	0.004	0.005
			(0.005)	(0.006)	(0.005)	(0.007)
Log social hours		0.054***		0.054***		0.053***
		(0.002)		(0.002)		(0.002)
Dummy for zero social hour		-0.137***		-0.142***		-0.141***
		(0.013)		(0.013)		(0.013)
Dummy for zero to one social hour		-0.057***		-0.062***		-0.057***
		(0.012)		(0.013)		(0.012)
Number of observations	117,302	72,836	116,752	72,559	115,566	71,777
Number of counties	3,004	2,903	3,004	2,903	3,001	2,899
Adjusted R-squared	0.037	0.075	0.031	0.071	0.038	0.076

Notes: The odd columns follow Eq (2) and the even columns follow Eq (3). Variables in the vector ${\text{X}}_{it}^{\prime }$ and state-year dummies are controlled in all models, but coefficients are not reported. The Standard errors in parentheses are clustered within counties. +, *, **, and *** indicate significance at the 10, 5, 1, 0.1% levels respectively.

From Model (1) in Table 6 we see that the weekend effect of happiness for full-time workers in high-trust workplaces is 0.025, while for those reporting non-trusting workplace environment it is 0.070, which equals to 0.025 plus the 0.045 coefficient on the interaction term. This implies that the weekend effect is almost three times larger for those working in a low-trust environment. Results in Model (3) using the alternative workplace environment variable confirm the finding: full-time workers reporting boss-like supervisor have weekend effects twice as do workers with partner-like supervisors. If we include both indicators of workplace social context, as shown in Model (5), the weekend effect of happiness for full-time workers reduces to 0.022, and that for unfavorable environment (both indicators equal to 1) rises to 0.073. Workers with a boss-like supervisor in a low trust workplace thus have weekend effects more than three times as large as for those having a partner-like supervisor in a higher trust work environment.

If we control also for the social time variable, the weekend effect for those full-time workers reporting favorable workplace environment is reduced to zero, as shown in Models (2) and (4), or even slightly negative (-0.003) as in Model (6). This suggests that weekend effects for respondents with favorable workplace environments are only due to the differing amounts of social time on weekends and weekdays. The weekend effects for those reporting unfavorable workplace environments are now 0.041, 0.039, and 0.034 in Models (2), (4) and (6) respectively, which are much smaller than the effects calculated without accounting for the difference in social time between weekends and weekdays.

We observe similar patterns for part-time workers for the weekend effect of happiness in Table 7. Without controlling for social time variable, the weekend effect for those reporting unfavorable workplace environments is always larger than for those reporting favorable environments (by about 0.013). Moreover, accounting for social time differences between weekends and weekdays reduces the weekend effect for those part-time workers reporting favorable workplace environment to a level not significantly different from zero.

The results for happiness in Tables 6 and 7 are summarized in Panel A of Table 8, in which we report weekend effects in different scenarios. To save space, we follow the format of Panel A to report the results for the remaining positive emotions in Panels B and C of Table 8, and for the four negative emotions in Panels A to D of Table 9.

**Table 8:** OLS Estimates of Weekend Effects for Positive Emotions.

	Full-time workers		Part-time workers	
	Not controlling social time	Controlling social time	Not controlling social time	Controlling social time
*Panel A*. *Dependent Variable*: *Happiness*				
Trusting workplace	0.025***	0.000	0.014***	-0.001
Non-trusting workplace	0.070***	0.041***	0.024***	0.017^+^
Partner-like supervisor	0.025***	-0.000	0.013***	-0.001
Boss-like supervisor	0.050***	0.025***	0.019***	0.008
Trusting workplace & partner-like supervisor	0.022***	-0.003*	0.013***	-0.002
Non-trusting workplace & boss-like supervisor	0.073***	0.045***	0.026***	0.020*
*Panel B*. *Dependent Variable*: *Enjoyment*				
Trusting workplace	0.048***	0.018***	0.029***	0.010***
Non-trusting workplace	0.133***	0.097***	0.051***	0.044***
Partner-like supervisor	0.048***	0.017***	0.028***	0.008*
Boss-like supervisor	0.097***	0.066***	0.042***	0.030***
Trusting workplace & partner-like supervisor	0.042***	0.011***	0.026***	0.006
Non-trusting workplace & boss-like supervisor	0.140***	0.103***	0.055***	0.048***
*Panel C*. *Dependent Variable*: *Laughter*				
Trusting workplace	0.026***	-0.004**	0.020***	-0.000
Non-trusting workplace	0.081***	0.043***	0.038***	0.020^+^
Partner-like supervisor	0.027***	-0.005**	0.018***	-0.001
Boss-like supervisor	0.057***	0.025***	0.031***	0.010
Trusting workplace & partner-like supervisor	0.023***	-0.008***	0.017***	-0.002
Non-trusting workplace & boss-like supervisor	0.085***	0.048***	0.043***	0.023*

Notes: Standard errors in parentheses are clustered within counties. +, *, **, and *** indicate significance at the 10, 5, 1, 0.1% levels respectively.

**Table 9:** OLS Estimates of Weekend Effects for Negative Emotions.

	Full-time workers		Part-time workers	
	Not controlling social time	Controlling social time	Not controlling social time	Controlling social time
*Panel A*. *Dependent Variable*: *Worry*				
Trusting workplace	-0.077***	-0.054***	-0.063***	-0.050***
Non-trusting workplace	-0.110***	-0.079***	-0.080***	-0.070***
Partner-like supervisor	-0.080***	-0.057***	-0.066***	-0.052***
Boss-like supervisor	-0.091***	-0.062***	-0.066***	-0.052***
Trusting workplace & partner-like supervisor	-0.078***	-0.056***	-0.059***	-0.051***
Non-trusting workplace & boss-like supervisor	-0.110***	-0.078***	-0.079***	-0.067***
*Panel B*. *Dependent Variable*: *Sadness*				
Trusting workplace	-0.010***	-0.000	-0.010***	-0.002
Non-trusting workplace	-0.032***	-0.018***	-0.031***	-0.017
Partner-like supervisor	-0.010***	0.000	-0.011***	-0.001
Boss-like supervisor	-0.023***	-0.010***	-0.018***	-0.009
Trusting workplace & partner-like supervisor	-0.008***	0.001	-0.010***	-0.001
Non-trusting workplace & boss-like supervisor	-0.034***	-0.019***	-0.033***	-0.021^+^
*Panel C*. *Dependent Variable*: *Anger*				
Trusting workplace	-0.029***	-0.021***	-0.011***	-0.012***
Non-trusting workplace	-0.085***	-0.074***	-0.026**	-0.033**
Partner-like supervisor	-0.030***	-0.021***	-0.013***	-0.015***
Boss-like supervisor	-0.058***	-0.050***	-0.015**	-0.014*
Trusting workplace & partner-like supervisor	-0.025***	-0.018***	-0.012***	-0.013***
Non-trusting workplace & boss-like supervisor	-0.088***	-0.077***	-0.026**	-0.031***
*Panel D*. *Dependent Variable*: *Stress*				
Trusting workplace	-0.158***	-0.123***	-0.087***	-0.073***
Non-trusting workplace	-0.201***	-0.163***	-0.100***	-0.086***
Partner-like supervisor	-0.162***	-0.126***	-0.093***	-0.078***
Boss-like supervisor	-0.179***	-0.142***	-0.079***	-0.067***
Trusting workplace & partner-like supervisor	-0.158***	-0.123***	-0.093***	-0.078***
Non-trusting workplace & boss-like supervisor	-0.202***	-0.164***	-0.095***	-0.081***

Notes: Standard errors in parentheses are clustered within counties. +, *, **, and *** indicate significance at the 10, 5, 1, 0.1% levels respectively.

The weekend effect for enjoyment is reported in Panel B of Table 8. Without controlling for the social time variable, the weekend effect for full-time workers is 0.042 for those reporting favorable workplace social contexts, compared to 0.140 for those in unfavorable workplace social contexts. Holding social time constant, the two corresponding values reduce to 0.011 and 0.103. Thus controlling for social time reduces the weekend effect by about three-quarters for those with good workplace environments, and by one-quarter for those with socially unfavorable workplaces. If we compare the weekend effect for those who report unfavorable workplace environments without accounting for social time, 0.140, with the effect, 0.011, for those who report favorable environments, after allowing for social time, the reduction is over 90%. The proportionate reduction is almost 90% (0.055 to 0.006) for part-time workers. In Panel C of Table 8 we summarize the weekend effects for laughter. We see that social time and the workplace social context together fully explain the weekend effect.

In the four panels of Table 9 we report the weekend effects for worry, sadness, anger, and stress respectively. Compared to our previous results for positive emotions, the explanatory power of workplace environment and social time is lower for all negative emotions except sadness. Specifically, in the case of full-time workers, social time and workplace environment together explain about half of the weekend effects for worry (-0.110 to -0.056), 80% for anger (-0.088 to -0.018) and 40% for stress (-0.202 to -0.123). For part-time workers the part of the weekend effect explained by the social context variables is one-third for worry (-0.079 to -0.051), 50% for anger (-0.026 to -0.013), and 20% for stress (-0.095 to -0.078).

**Fig 2:**
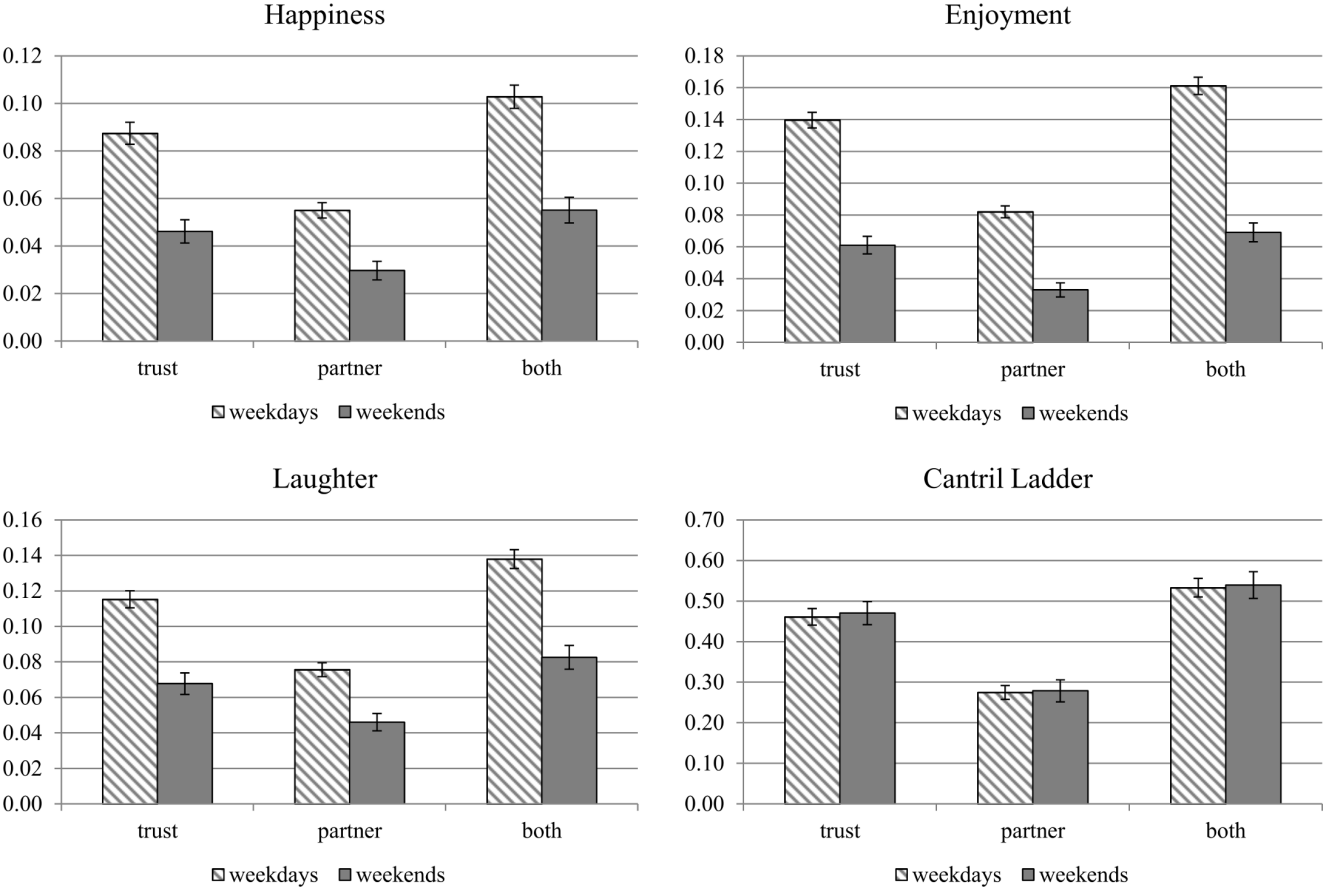
Impacts of Workplace Environment on Positive Emotions and Life Evaluations on Weekends and Weekdays—Full-time Workers. Notes: The vertical bars indicated by “trust” show the difference in positive emotions or life evaluations between those reporting “trusting workplace” and those reporting “non-trusting workplace”. The bars indicated by “partner” show the difference between those reporting “partner-like supervisor” and those reporting “boss-like supervisor”. The bars indicated by “both” show the difference between those reporting both “trusting workplace” and “partner-like supervisor” and those reporting “non-trusting workplace” and “boss-like supervisor”. 95% confidence interval is also shown for each bar.

**Fig 3:**
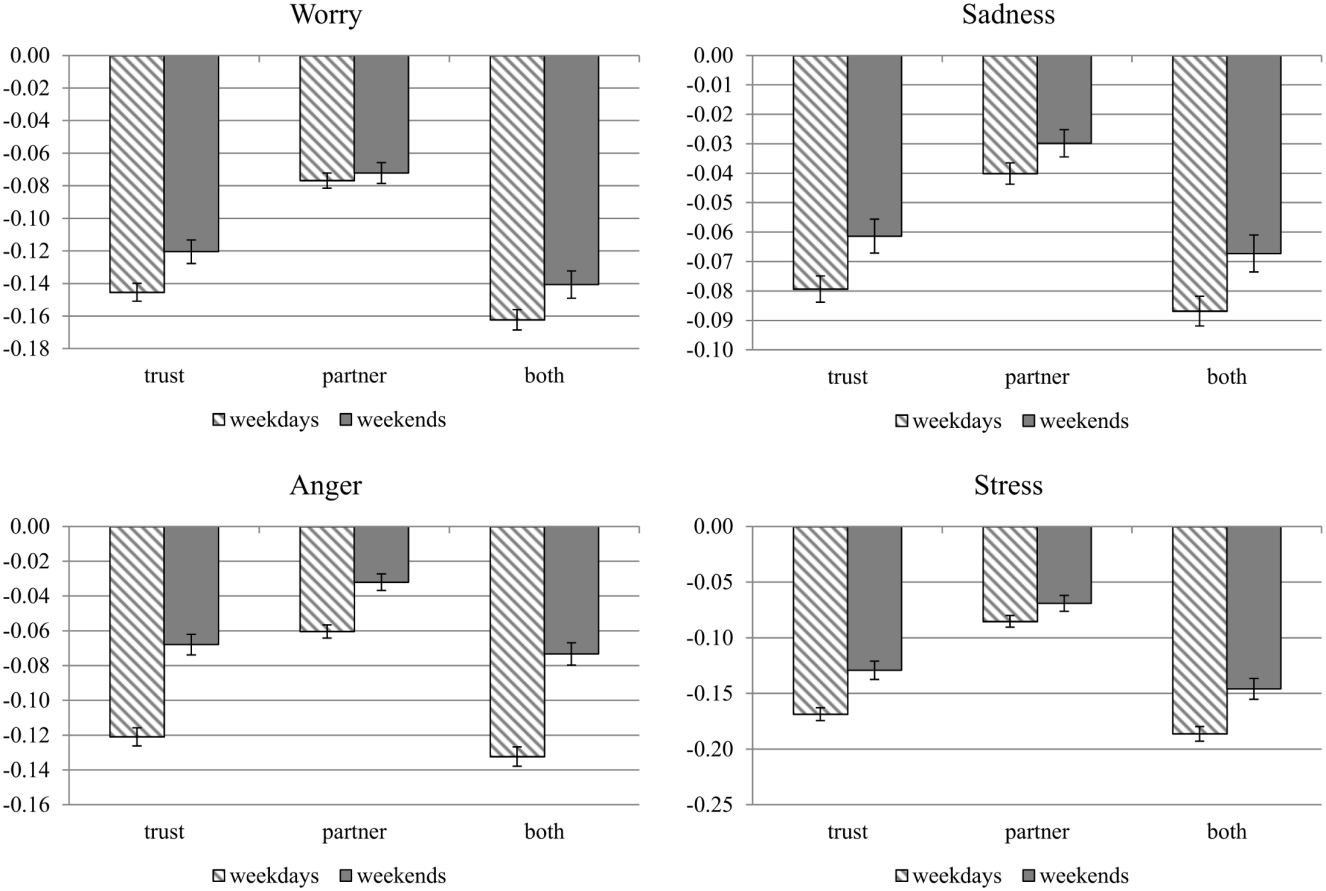
Impacts of Workplace Environment on Negative Emotions on Weekends and Weekdays—Full-time Workers. Notes: The vertical bars indicated by “trust” show the difference in negative emotions between those reporting “trusting workplace” and those reporting “non-trusting workplace”. The bars indicated by “partner” show the difference between those reporting “partner-like supervisor” and those reporting “boss-like supervisor”. The bars indicated by “both” show the difference between those reporting both “trusting workplace” and “partner-like supervisor” and those reporting “non-trusting workplace” and “boss-like supervisor”. 95% confidence interval is also shown for each bar.

If weekends are better than weekdays for those with poor social contexts in their workplaces, could bad jobs provide a way of getting better weekends? The answer is “no”. Taking “happiness” as an example, we can see this point clearly from Table 6, which shows that the coefficients of “dummy for non-trusting workplace” in all columns are negative and statistically significant. We highlight in Figs 2 and 3 our evidence showing clearly that full-time workers in jobs with good social contexts have more positive emotions, fewer negative emotions, and higher life evaluations on both weekends and weekdays. Fig 2 shows the results for three positive emotions and life evaluations. The workplace impacts on happiness on weekends and weekdays indicated by “trust” can be calculated from the coefficient of “dummy for non-trusting workplace” and “dummy for non-trusting workplace*weekend” in column (2) of Table 6. Similarly, the impacts indicated by “supervisor” can be calculated by “dummy for boss-like supervisor” and “dummy for boss-like supervisor*weekend” in column (4), and the impacts indicated by “both” are from the all these four variables in column (6). The results for other emotions and life evaluations are taken from parallel regressions reported in the online appendix. For all three emotions, the gains from a good workplace social context are significantly greater on weekdays than on weekends, but weekend emotions are nonetheless heavily dependent on the memories and anticipations of what has and will happen during the working week. Some of these effects may be due to the fact that many respondents work on weekends, but our sample of employed respondents is not asked about their days of work, beyond whether they work part-time or full-time.

Fig 3 shows the results for four negative emotions. Consistent with the results for positive emotions, the gains from a good workplace social context are significantly greater on weekdays than on weekends. The corresponding figures for part-time workers, reported in an online appendix show results consistently smaller, as we have already seen in Tables 6–9, than those for full-time workers.

Life evaluations are heavily influenced by the social context at work, but to an amount that is the same on weekdays and weekends. When people are asked about their lives as a whole, what goes on at work is clearly a very important determinant of their answers. Since they are being asked about their lives as a whole, and not about the previous day, their answers do not depend, and nor should they, on the day of the week when the question was asked. The results at the bottom of Table 2 show nonetheless for the employed population as a whole that overall life evaluations are slightly but significantly higher on weekends than on weekdays, by an amount equal to about 0.2%, a tiny fraction of the difference shown for any of the emotions, which is consistent with [2]. This finding helps to validate both emotional reports and life evaluations, since the former are intended to reveal day-to-day changes, while the latter are intended to look beyond the day-to-day variations in experiences to provide a broader measure of subjective well-being.

## Conclusions

In this paper we estimate the size and sources of weekend effects for seven emotions for the working population in the United States, using the Gallup/Healthways US Daily Poll 2008–2012. We first find that weekend effects, measured as the difference in fractions of the population reporting each emotion between weekends and weekdays, are statistically and economically significant for all seven emotions. Moreover, full-time workers have larger weekend effects than do part-time workers for all emotions except for sadness, where the effects are similar for full-time and part-time workers. We also show that the weekend effects we find are not driven by the bias of self-selection into weekends versus weekdays.

We then explore the sources of weekend effects and find that the social quality of the workplace is a key correlate of emotions and life evaluations for US employed workers on all days of the week, positive for positive emotions and life evaluations and negative for negative emotions. Moreover, we find that the effects of workplace quality are larger on weekdays than on weekends. This also means that the weekend effects are much smaller for workers with good workplace social contexts, as indicated by high workplace trust and a partner-like boss. Moreover, social time can largely or even entirely explain the remaining weekend effects for positive emotions and sadness for workers with favorable workplace social contexts. The workplace social environment and social time together almost completely account for the weekend effects for happiness, laughter, enjoyment and sadness, for both full-time and part-time workers. The explanatory power is lower for the remaining negative emotions. Taken together, the quality of the social contexts on and off the job are the primary forces behind weekend effects in the subjective well-being of the working population of the United States.
